# Editorial

**DOI:** 10.1120/jacmp.v4i3.2513

**Published:** 2003-06-01

By this third issue from Minnesota we hopefully have it down right. In the last Editorial I mentioned that John Horton was rotating off the Editorial Board, as he is Chair of JACMP. We have added Mark J. Rivard to the Editorial Board. Mark, based in Boston, is very active in the TG‐43 rewrite and was just the person we needed. Again I thank our Editorial Board members for their selfless service as well as their suffering under prodding from the Manuscript Manager as well as the Editor‐in‐Chief. We try to get as many high quality papers published each quarter as possible. Our reject rate is in excess of 30 percent, indicating that real peer reviewing is going on.

Once again it is time to announce the awards for the best papers published in JACMP in Volume 3 (2002). This year the awards were selected by the Editorial Board (excluding any who were authors). The awards were presented in Lake George, NY. The authors of each paper collectively receive a $500 check, and each author receives a certificate. The Journal is grateful to LAP of America, Radiological Imaging Technology, Elekta, and PTW New York (new this year) for supporting the recognition of outstanding papers in JACMP.

The LAP Award for Excellence for the best radiation oncology paper went to Kent A. Gifford, David S. Followill, H. Helen Liu, and George Starkschall for “Verification of the accuracy of a photon dose‐calculation algorithm,” JACMP, Vol. 3, No.1, Winter 2002.

The RIT Award for Excellence for the best diagnostic imaging paper went to Thomas R. LaVoy, Walter Huda, and Kent M. Ogden for “Radiographic techniques in screen‐film mammography,” JACMP, Vol. 3, No. 3, Summer 2002.

The Elekta Award for Excellence for the best professional medical physics paper went to W. S. Bice, Jr., E. S. Walker, S. Gearty, A. V. Walker, J. R. Marbach, and B. R. Prestidge for “A comparative evaluation of loading times and exposures for permanent prostate brachytherapy,” JACMP, Vol. 3, No. 4, Fall 2002.

The PTW Award for Excellence for the best basic dosimetry paper to M. A. MacKenzie, M. Lachaine, B. Murray, B. G.Fallone, D. Robinson, and G. C. Field for “Dosimetric verification of inverse planned step and shoot multileaf collimator fields from a commercial treatment planning system,” JACMP, Vol. 3, No. 2, Spring 2002.

Over the course of the past year it has become painfully clear that JACMP cannot be sustained by ACMP and that the original business model was never realized and if implemented (paid subscriptions) would fall short. In the last year or so, two major benefactors have kept JACMP afloat. With this, an ad hoc ACMP/AAPM committee was formed to consider how the continuation of publishing clinical material could be done. By the time this issue of JACMP is available online, the AAPM Board will be preparing to or will have considered a proposal. I will not jinx the process by saying too much except to thank the AAPM members (Boone, Marshall, Orton, and Martin) who have worked with ACMP members (Sternick, Smathers, and McCullough) to craft what might be a reasonable way to proceed. Enough teasing for the time being…

I look forward to bringing you the next Editorial in Volume 4. Hopefully at that time I will be able to bring you up to date on some additional exciting issues. In the meantime, please feel free to contact me (ecmc@mayo.edu) about any issues related to JACMP. And send us your clinically related manuscripts. The following members of the Editorial Board would like nothing better than to review your contributions.

Edwin C. McCullough

Editor‐in‐Chief

